# Polymer-free dual drug-eluting stents evaluated in a porcine model

**DOI:** 10.1186/s12872-017-0654-7

**Published:** 2017-08-15

**Authors:** Bin Zhang, Bo Zheng, Xingang Wang, Qiuping Shi, Jia Jia, Yong Huo, Chunshui Pan, Jingyan Han, Ming Chen

**Affiliations:** 1 Department of Cardiology, Peking University First Hospital, No. 8 Xishiku Str., Xicheng District, Beijing, 100034 China; 2 Department of Integration of Chinese and Western Medicine, School of Basic Medical Sciences, Peking University, No. 38 Xueyuan Rd, Haidian District, Beijing, 100191 China; 3 Tasly Microcirculation Research Center, Peking University Health Science Center, Beijing, 100191 China

**Keywords:** Stent thrombosis, Endothelialization, Probucol, Optical coherence tomography, Porcine

## Abstract

**Background:**

Although drug-eluting stents have dramatically reduced the rates of restenosis and target lesion revascularization, they are associated with stent thrombosis (ST), a catastrophic event likely due to delayed endothelialization and chronic inflammation caused by the polymer and the metal scaffolds. To increase the safety and efficacy of stents, polymer-free dual drug-eluting stents (DDES) have been developed.

**Methods:**

A total 160 stents (Bare-metal stents (BMS), polymer-free probucol stents (PrES), sirolimus-eluting stents (SES) and DDES) were randomly implanted in the coronary arteries of 80 pigs. 14, 28, 90 and 191 days after implantation, QCA and OCT evaluations were performed in 20 pigs respectively, and the arteries were harvested for scanning electron microscope (SEM), histomorphology, histopathology analyses and for the relative expression of CD31, CD34 and CD133 on mRNA and protein levels.

**Results:**

At the 14-day time point, there were significant differences in the strut rate coverage (*p* = 0.011), with greater coverage in the PrES than in the SES group (53.2%vs. 20.3%, *p* = 0.002). As well, there were no significant differences in the expression of CD31, CD34 and CD133 between groups in mRNA and protein level.

**Conclusions:**

DDES were as safe as BMS and SES, but they did not further improve the endothelialization of the stented coronary arteries in the porcine model.

## Background

Coronary heart disease is one of the leading causes of mortality and morbidity worldwide. Noninvasive percutaneous coronary intervention (PCI) with stenting has become the standard of care, but delayed endothelialization and chronic inflammation caused by the polymer and the metal scaffolds lead to the development of stent restenosis (SR) and stent thrombosis (ST), the most important clinical problems following PCI. In an effort to decrease the incidence and consequences of these complications, bioresorbable coronary scaffolds have been developed albeit their use did not further improve short-term outcomes [1, 2].Polymer-free dual drug-eluting stents (DDES) may be a more effective approach.

Sirolimus (rapamycin) and its derivatives are still the most used antiproliferative agents in contemporary drug-eluting stents despite the fact they are associated with delayed endothelialization of the stents because of the inhibition of endothelial cell (EC) and endothelial progenitor cell (EPC) proliferation and differentiation [3, 4]. Indeed, autopsy studies have shown that the best morphometric predictor of late ST was the ratio of uncovered struts relative to the total stent struts, which is a marker of stent endothelialization [5]. Probucol is not only a potent antioxidant and lipid-lowering drug, it is also effective in reducing the risk of restenosis and the incidence of major adverse cardiac events after PCI [6]. Indeed, several studies, both in animals and humans, have shown some improvement in the rate of endothelialization of the stented arteries by probucol [7–9]. However, few preclinical studies have focused on the endothelialization of stented arteries associated with novel polymer-free DDESs. Consequently, the aim of the present study was to evaluate the efficacy and safety of the polymer-free stent-based delivery system of probucol alone compared with rapamycin, and probucol in combination with rapamycin in a porcine model of coronary artery stenting.

## Methods

### Stents

The stents platform and drug loading were described previously [10]. Four types of stents were used in this study shared the same metallic platform: 316 L stainless steel bare-metal stents (BMS), polymer-free probucol-eluting stents (PrES) loaded with 0.8 μg/mm^2^ probucol, polymer-free sirolimus-eluting stents (SES) loaded with 1.6 μg/mm^2^ sirolimus and the novel polymer-free DDES coated with 1.6 μg/mm^2^ sirolimus and 0.8 μg/mm^2^ probucol. All stents were purchased from Lepu Medical (Beijing, China).

### Animals

The study was approved by the Institutional Animal Care and Use Committee of Peking University First Hospital, and conformed to the National Institutes of Health Guide for the Care and Use of Laboratory Animals. Chinese miniature swines of either sex (25 kg to 35 kg, 6 to 8 months of age) were provided by the China Agricultural University. All animals were fed a standard laboratory chow diet without lipid throughout the course of the study. In order to decrease the incidence of acute thrombosis, premedication with 300 mg aspirin (Bayer, Germany) and 75 mg clopidogrel (Xin Li Tai Pharmaceutical Co Ltd., China) was administered before stent implantation for one day. Aspirin (100 mg/d) and clopidogrel (75 mg/d) were then given until sacrificed.

### Stent implantation and experimental groups

A total of 160 stents (BMS, PrES, SES and DDES) were randomly implanted in the left anterior descending, circumflex or right coronary arteries of 80 pigs (two types of stents per two arteries per pig) (Table 1). The anesthesia and stent implantation procedures have been described previously [11].

**Table 1:** Stents distribution in four endpoints included in the analysis

	14 day			28 day			90 day			191 day		
	LAD	LCX	RCA	LAD	LCX	RCA	LAD	LCX	RCA	LAD	LCX	RCA
BMS	7	2	0	3	5	1	5	3	0	4	1	4
PrES	5	5	0	5	4	0	3	4	1	4	5	0
SES	2	5	2	5	2	1	3	3	2	5	4	0
DDES	5	4	1	4	4	0	5	3	0	5	1	3

*BMS* Bare-Metal Stents, *PrES* Probucol-Eluting Stents, *SES* Sirolimus-Eluting Stents, *DDES* Dual Drug-Eluting Stents, *LAD* Left Anterior Descending, *LCX* Left Circumflex, *RCA* Right Coronary Arteries

### Quantitative coronary angiography (QCA) evaluation

At 14, 28, 90 and 180 days following stent implantation, repeat QCA were performed in 20 animals using the CAAS 5.9 QCA Software respectively (PIEMEDICAL IMAGINE, The Netherlands). Coronary artery measurements included baseline vessel diameter, minimal lumen diameter immediately after implantation (I-MLD), reference vessel diameter (RVD), MLD at repeat angiography (R-MLD) and lumen loss (LL).

### Optical coherence tomography (OCT) imaging and evaluation

The OCT evaluation of the stented vessels was performed 14 and 28 days immediately after the repeat QCA (*n* = 7 in each group) at a pullback speed of 2.5 cm/s using a commercially available OCT system (C7-XR Dragonfly System, St. Jude Medical, USA). Cross-sectional OCT images were analyzed by frame measurements. Lumen area, internal elastic lamina area, total struts and covered struts were assessed. Neointimal area (NA) and the ratio of covered struts (CSR) were then calculated [5].

### Histopathological evaluation

At 14, 28, 90 and 180-days following stent implantation, twenty animals in each group were sacrificed, and the hearts were removed and perfused with heparin saline for five minutes at a pressure of 100 mmHg (1 mmHg = 0.0133 kPa). The stented vessel segments were separated into three pieces on ice, and the segment with the higher restenosis rate (according to the QCA results) was fixed with 10% formaldehyde solution and embedded in methyl methacrylate plastic prepared by the Peking University School of Stomatology for histological examination [11]. The other segments were stored in freezing tubes and stored at −80 °C for subsequent molecular biology study (western blot and real time-polymerase chain reaction (PCR)).

### Scanning electron microscopy (SEM) image acquisition and evaluation

Scanning electron microscopy was performed on longitudinal sections harvested 14 and 28 days following the OCT imaging. The hearts were removed and perfused as described above. The stented vessel segments were separated and fixed with 3% buffered glutaraldehyde and 1% buffered osmium tetroxide (*n* = 1 for each group). The samples were then dehydrated in a series of ethanol baths (50%, 75% and 100%), dried in liquid CO_2_ in a critical point dryer (72.8 atm, 31 °C), and sputter-coated for 3 min at 15 mA with Au. En face SEM images (H-450, Hitachi, Japan) were acquired at low magnification (×18 magnification) to evaluate the overall neointimal coverage of the stents, and at various high magnifications to identify the composition of the tissue covering the surfaces of the stents. Endothelial cells were identified as sheets of closely connected monolayer cells with a spindle or polygonal shape [12].

### Western blot and real time PCR evaluation

All the samples stored for the molecular biology study were divided into two parts, one to extract the total protein content for Western blot analysis and the other to extract the total RNA content for Real time PCR. At the four time points (14, 28, 90 and 180 days following stent implantation), the stented segments of all stent types were evaluated for the expression of CD31, CD34 and CD133. Protein extracts were fractionated on SDS ployacrylamide gels, transferred to PVDF membranes, incubated with a purified affinity polyclonal antibody to CD31 (Earthox: E021010, USA), CD34 (Santa Cruz Biotechnology: ab80508, USA), CD133 (Abcam: ab109366, USA) and glyceraldehyde-3-phosphate dehydrogenase (GAPDH) (Earthox: E021010, USA), washed and incubated with a second antibody. Signals were detected using ECL chemiluminesence detection system, and autoradiographic signal were quantified by densitometry. The relative expression level of the markers was determined by the comparison of band intensities on bolts to GAPDH, a housekeeping protein employed as an internal control. Total RNA of each stented segment was isolated using an RNA extraction Kit (Roche: 12,033,674,001, Germany).

Total cDNA, synthesized by reverse transcription from total RNA using Reverse Transcription Kit (Roche: 05081955001, Germany), was amplified by PCR for 35 cycles at 50 °C for 2 min, 95 °C for 10 min, and 58 °C for 1 min using SYBR Green/ROX qPCR Master Mix (Roche: 06924204001, Germany) on Fluorescent Quantitative PCR (Opticon 2 MJ Research, Bio-Rad, USA). The PCR primer sets for CD31 (5′-CACCGAGGTCTGGGAACAAA-3′ and 5′-CTGCGGTCCTAAGTCCCATC-3′), CD34 (5′-GTCTTGGCCAACGGAACAGA-3’and 5′-GTCTTCGCCCAGCCTTTCTC-3′), CD133 (5′-AATGCCTCTGGTGGGGCTTA-3′ and 5′-CACCAAGAGGGAAACGGCAA-3′) and GAPDH (5′-CGATGGTGAAGGTCGGAGTG-3′ and 5′-TGCCGTGGGTGGAATCATAC-3′) were used for amplification.

### Statistical analysis

Values in normal distribution were expressed as mean ± standard deviation and non-normal distributions were expressed as median and interquartile range. Group imaging, histological and integral data were analyzed with a one-way ANOVA test (normal distribution values) or Kruskal-Wallis rank test (non-normal distribution values). N represents the number of stents of different types or samples. Comparisons within and between groups were performed using paired t test (normal distribution values) and Wilcoxon’s signed-rank test (non-normal distribution values). The value of *p* < 0.05 was considered statistically significant. All statistical analyses were performed with SPSS 19.0 system software (IBM, USA).

## Results

A total of 160 stents were successfully implanted in the coronary arteries of 80 pigs. Overall, 7 animals died during the course of the study; 2 of respiratory failure (1 in the 14-day group and 1 in the 28-day group) which was most likely due to the anesthesia, 3 of post-operative puncture site bleeding (2 in the 28-day group and 1 in the 90-day group), 1 of pneumonia (at 71 days follow-up) and 1 of ileus (at 180 days follow-up). None of the animals showed any evidence of ST or myocardial infarction. The latter 2 animals were included in the histological analysis. All other animals survived the intended study follow-up period without any angiographic ST or clinical complications. Stents distribution included in the analysis was showed in Table 1.

### Quantitative coronary angiography evaluation

**Fig. 1:**
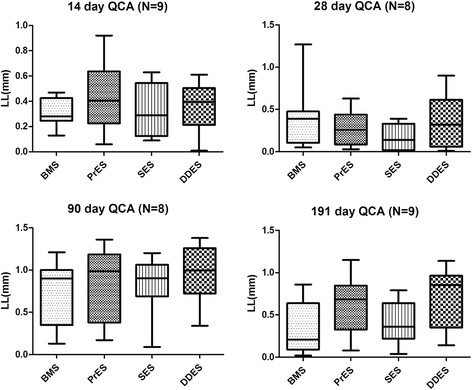
Lumen loss (LL) at all four endpoints. QCA: Quantitative Coronary Angiography; LL: Lumen Loss; BMS: Bare-Metal Stents; PrES: Probucol-Eluting Stents; SES: Sirolimus-Eluting Stents; DDES: Dual Drug-Eluting Stents

From baseline, the RVD, expansion ratio and the LL remained similar among the groups at all the four endpoints (Fig. 1 and Table 2).

**Table 2:** Quantitative coronary angiography evaluation at all the four endpoints

		RVD (mm)	P	ER	P	LL (mm)	P	DS%	P
14 day									
	BMS (*n* = 9)	2.60 ± 0.22	0.061	1.13 ± 0.14	0.067	0.32 ± 0.11	0.655	12.43 ± 4.50	0.429
	PrES (*n* = 10)	2.41 ± 0.31		1.25 ± 0.17		0.43 ± 0.29		19.12 ± 14.87	
	SES (*n* = 9)	2.62 ± 0.28		1.17 ± 0.08		0.34 ± 0.21		12.99 ± 7.61	
	DDES(*n* = 10)	2.36 ± 0.16		1.27 ± 0.09		0.36 ± 0.18		15.46 ± 8.07	
28 day									
	BMS (*n* = 8)	2.55 ± 0.39	0.994	1.19 ± 0.19	0.946	0.41 ± 0.39	0.409	18.26 ± 20.64	0.365
	PrES (*n* = 8)	2.56 ± 0.31		1.16 ± 0.09		0.28 ± 0.21		10.34 ± 6.93	
	SES (*n* = 8)	2.55 ± 0.19		1.18 ± 0.12		0.18 ± 0.15		6.94 ± 5.76	
	DDES(*n* = 8)	2.52 ± 0.38		1.20 ± 0.14		0.37 ± 0.32		15.90 ± 15.14	
90 day									
	BMS (*n* = 8)	2.37 ± 0.30	0.945	1.26 ± 0.13	0.662	0.76 ± 0.36	0.767	32.71 ± 16.40	0.809
	PrES (*n* = 8)	2.30 ± 0.22		1.29 ± 0.08		0.85 ± 0.44		37.19 ± 18.71	
	SES (*n* = 8)	2.30 ± 0.27		1.29 ± 0.10		0.83 ± 0.34		35.45 ± 15.47	
	DDES(*n* = 8)	2.34 ± 0.18		1.24 ± 0.10		0.96 ± 0.35		41.66 ± 15.79	
191 day									
	BMS (*n* = 9)	2.41 ± 0.30	0.335	1.28 ± 0.16	0.368	0.34 ± 0.31	0.079	15.76 ± 16.68	0.811
	PrES (*n* = 9)	2.31 ± 0.29		1.24 ± 0.14		0.62 ± 0.34		27.44 ± 16.22	
	SES (*n* = 9)	2.31 ± 0.19		1.30 ± 0.10		0.41 ± 0.25		18.01 ± 11.28	
	DDES(*n* = 9)	2.52 ± 0.31		1.19 ± 0.11		0.72 ± 0.36		29.70 ± 15.11	

*RVD* reference vessel diameter, *ER* expansion ratio, *LL* lumen loss, *DS%* diameter stenosis %, *BMS* Bare-Metal Stents, *PrES* Probucol-Eluting Stents, *SES* Sirolimus-Eluting Stents, *DDES* Dual Drug-Eluting Stents

### Optical coherence tomography evaluation

**Fig. 2:**
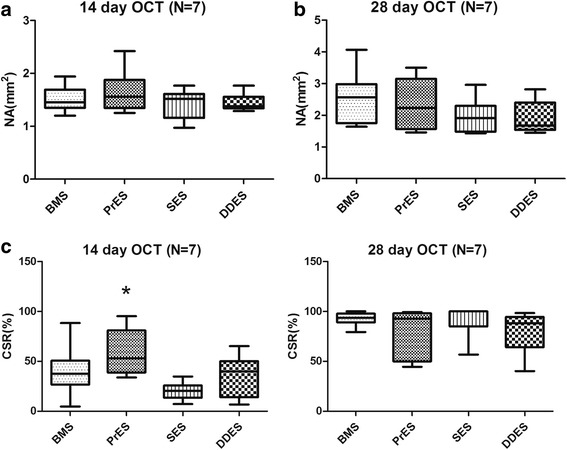
**a** Neointimal area (NA) at 14-day and 28-day by optical coherence tomography. OCT: Optical Coherence Tomography; NA: Neointimal Area; BMS: Bare-Metal Stents; PrES: Probucol-Eluting Stents; SES: Sirolimus-Eluting Stents; DDES: Dual Drug-Eluting Stents. **b** Covered struts rate (CSR) at 14-day and 28-day by optical coherence tomography. *represents CSR in PrES group (53.2%, interquartile: 39.0%–81.0%) was statistically higher than in the SES group (20.3%, interquartile: 13.5%–26.0%), (*P* = 0.002). OCT: Optical Coherence Tomography; CSR: Covered Struts Rate; BMS: Bare-Metal Stents; PrES: Probucol-Eluting Stents; SES: Sirolimus-Eluting Stents; DDES: Dual Drug-Eluting Stents

At 14- and 28-day time points, there were no significant differences in the NA between the four groups (Fig. 2a,b and Table 3), with no obvious ST, stent malapposition, dissection or prolapsed. At the 14-day time point, there was significant difference in the CSR between the four groups (*p* = 0.011), the CSR in the PrES group was greater than in SES group (53.2%, interquartile: 39.0% -81.0% vs. 20.3%, interquartile: 13.5% -26.0%, *p* = 0.002) (Fig. 2c and Table 3). However, a similar tendency was not observed at the 28-day time point.

**Table 3:** Optical Coherence Tomography Evaluation at 14 and 28-day

		NA (mm^2^)	P	CSR%	P
14 day					
	BMS (*n* = 8)	1.46 (1.35,1.69)	0.708	37.67 (26.74,50.65)	0.011
	PrES (*n* = 9)	1.56 (1.35,1.88)		53.24 (38.99,81.05)	
	SES (*n* = 7)	1.52 (1.16,1.61)		20.27 (13.49,25.95)	
	DDES(*n* = 8)	1.38 (1.34,1.56)		40.06 (14.12,50.16)	
28 day					
	BMS (*n* = 7)	2.57 (1.75,2.98)	0.300	93.64 (89.07,97.79)	0.644
	PrES (*n* = 7)	2.23 (1.57,3.15)		92.77 (50.00,98.23)	
	SES (*n* = 7)	1.91 (1.49,2.30)		85.15 (84.94,100.00)	
	DDES(*n* = 6)	1.68 (1.54,2.40)		88.22 (64.24,94.56)	

*NA* Neointimal Area, *CSR%* Ratio of Covered Struts, *BMS* Bare-Metal Stents, *PrES* Probucol-Eluting Stents, *SES* Sirolimus-Eluting Stents, *DDES* Dual Drug-Eluting Stents

### Histology evaluation

**Fig. 3:**
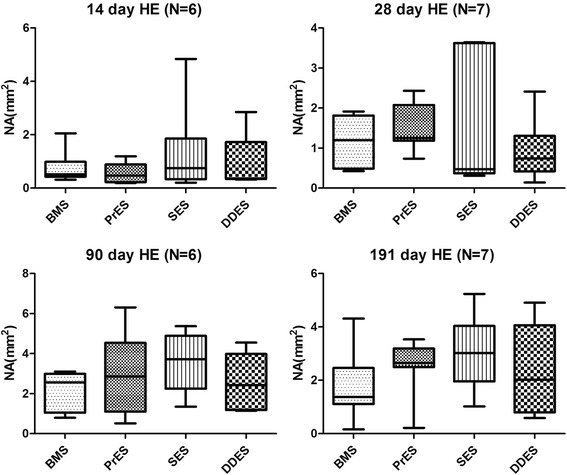
Neointimal area (NA) at 14-day, 28-day, 90-day and 191-day by hematoxylin and eosin staining. NA: Neointimal Area; BMS: Bare-Metal Stents; PrES: Probucol-Eluting Stents; SES: Sirolimus-Eluting Stents; DDES: Dual Drug-Eluting Stents

At the four time points, there were no significant differences in the NA, and injury, inflammation and endothelialization scores between the four groups (Fig. 3).

### Scanning electron microscope evaluation

**Fig. 4:**
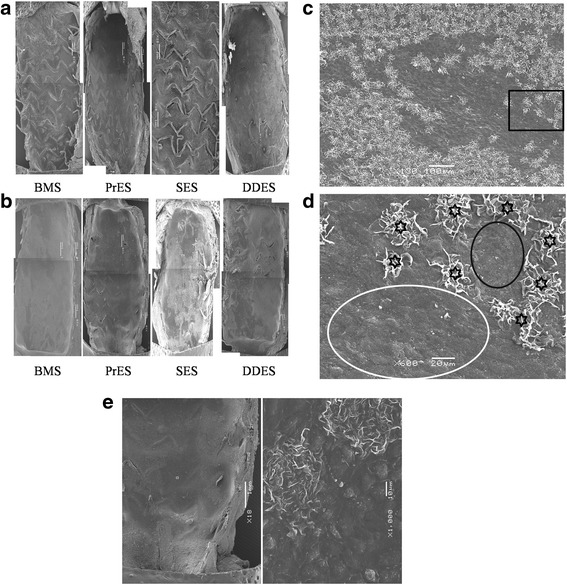
**a** Scanning electron microscopy (SEM) image at 14-day (×18). BMS: Bare-Metal Stents; PrES: Probucol-Eluting Stents; SES: Sirolimus-Eluting Stents; DDES: Dual Drug-Eluting Stents. **b** Scanning electron microscopy (SEM) image at 28-day (×18). BMS: Bare-Metal Stents; PrES: Probucol-Eluting Stents; SES: Sirolimus-Eluting Stents; DDES: Dual Drug-Eluting Stents. **c** Scanning electron microscopy (SEM) image at 28-day in the BMS group (×130). *Black rectangle* represents the region of **d**. **d** Scanning electron microscopy (SEM) image at 28-day in BMS group (×600). *Black circle* represents smooth muscle cells without extracellular matrix and endothelial cells. *White circle* represents the site completely covered by endothelial cells. Hexagrams represent extracellular matrix. **e** Scanning electron microscopy (SEM) image at 28-day in the PrES group (×18 left) and ×1000 right). *White rectangles* in the *left* image represent the area of the *right* image that is covered by endothelial cells, except for a small amount of extracellular matrix

At the 14- and 28-day time points, the rate of covered struts was observed at low magnification and the rate of covered struts by endothelial cells was observed at high magnification. The former was ranked as PrES > DDES > BMS > SES, and the latter was ranked as PrES > BMS > DDES > SES at 14 days (Fig. 4a). At 28 days, the rate of covered struts observed at low magnification was ranked as BMS > SES > PrES > DDES, while it was ranked as PrES > DDES > SES > BMS at high magnification (Fig. 4b).

### Western blot and real time polymerase chain reaction

**Fig. 5:**
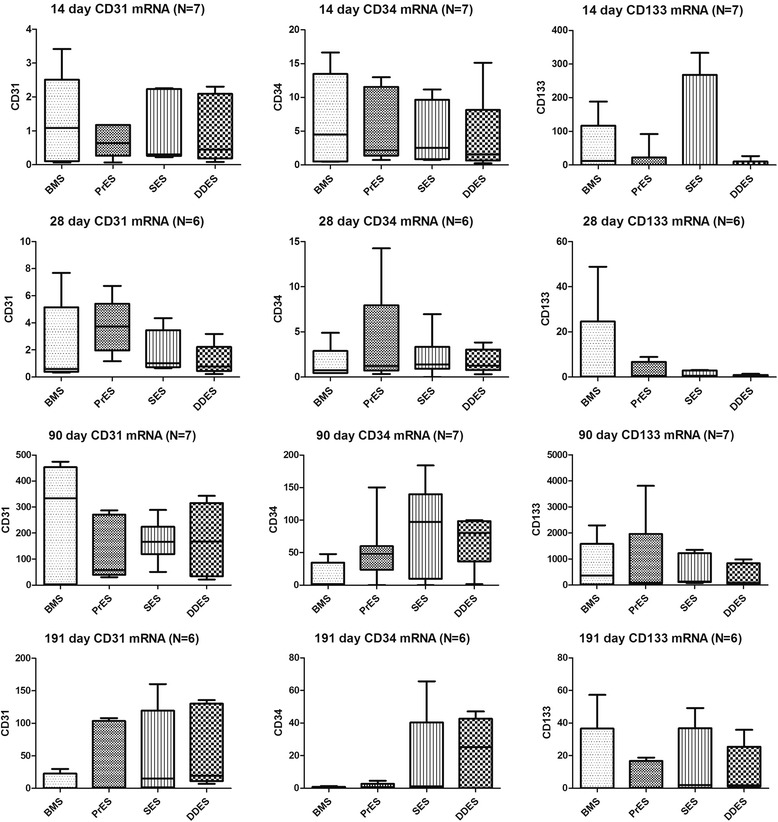
mRNA levels of CD31,CD34 and CD133 at 14-day, 28-day, 90-day and 191-day. BMS: Bare-Metal Stents; PrES: Probucol-Eluting Stents; SES: Sirolimus-Eluting Stents; DDES: Dual Drug-Eluting Stents

**Fig. 6:**
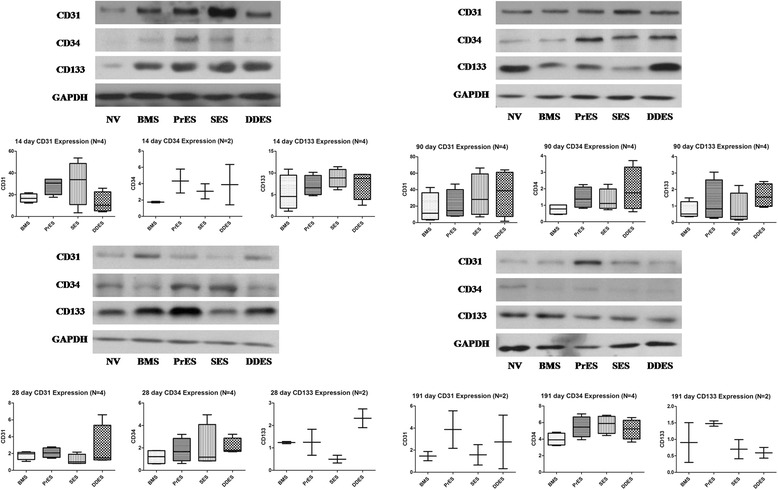
Protein expression of CD31, CD34 and CD133 at 14-day, 28-day, 90-day and 191-day. BMS: Bare-Metal Stents; PrES: Probucol-Eluting Stents; SES: Sirolimus-Eluting Stents; DDES: Dual Drug-Eluting Stents; NV: Normal Vessel

At the four time points, there were no significant differences in the expression of CD31, CD34 and CD133 between the four groups in terms of mRNA (Fig. 5) and protein level (Fig. 6).

## Discussion

Improvements in metal scaffolds, polymers and antiproliferative drugs of second generation DES have resulted in increased biocompatibility. However, they are still associated with a high incidence of SR and ST and, although polymer-free DES has been shown to be safe and efficacious in both animals [13–15] and humans [16–21], they are not routinely used in contemporary clinical practice. Animals study can effectively evaluate the safety of stents, but not their effectiveness. Clinical trials conducted over the past years were not large enough and lacked control groups to conclusively demonstrate the efficacy of polymer-free DES. Because of this, interventional cardiologists are often reluctant to use them despite their potential benefits. There are a few studies that have demonstrated that the antiproliferative drugs contained in the polymers could theoretically be lost in the process of transportation and release, reducing their long-term safety and short-term effectiveness. A coating of rapamycin or its derivatives on the surface of stents greatly reduces the rate of SR and target vessel revascularization, but not the occurrence of ST, especially late events. Because delayed endothelialization is believe to play a key role in the occurrence of late ST, research has attempted to improve the stent platform and the drug loading as well as drugs that may improve stent endothelialization by virtue of their antioxidant and anti-inflammatory properties. Probucol is such a drug, and several large clinical studies showed the non-inferiority of the polymer-free probucol/rapamycin DDES compared with the first and second generation polymer-based DES on the efficacy and safety [22–24]. However, a multi-center clinical study conducted in China showed that the same stent was associated with a higher incidence of restenosis and target vessels revascularization than the biodegradable polymer DES used as controls [25]. On the other hand, an animal study showed that succinobucol (a probucol analogue) eluting stents significantly increased the neointimal area of the stented vessel segment compared with rapamycin-eluting stents [26]. Thus, the efficacy and safety of this type of stent need to be further evaluated. The goal of the present study was to evaluate the safety of this novel DDES using a porcine model of coronary stenting. As well, we also evaluated whether the DDES could improve stent endothelialization.

Results of our study show that, irrespective of stent type, all animals had similar expansion ratio and injury scores at each time points, reflecting comparable vessel injury. Also, the lumen loss evaluated by QCA, the neointimal area evaluated by OCT and the histomorphology analysis assessing the neointimal proliferation level of the stented vessel segment were similar between all animals at all time points. On the one hand, our results show that the novel DDES has a similar anti-proliferative capability with the commercially available SES. However, the SES and DDES groups showed a similar anti-proliferative capability with the BMS group in each time point. This result may be due to the small sample size of each group.

Delayed endothelialization is considered to be a major underlying mechanism of ST, and numerous studies have evaluated the strut coverage level as a measure of reendothelialization. However, it is important to note that the struts that were completely covered did not represent the EC covering the entire lumen. These OCT studies could not distinguish if the coverage of the stent struts was by EC or other cell type. The coverage might not have the functionality of EC, especially its anti-thrombotic property [27]. Although some study has suggested that the strut coverage level might be associated with endothelial function [28], the evidence presented is unclear.

**Fig. 7:**
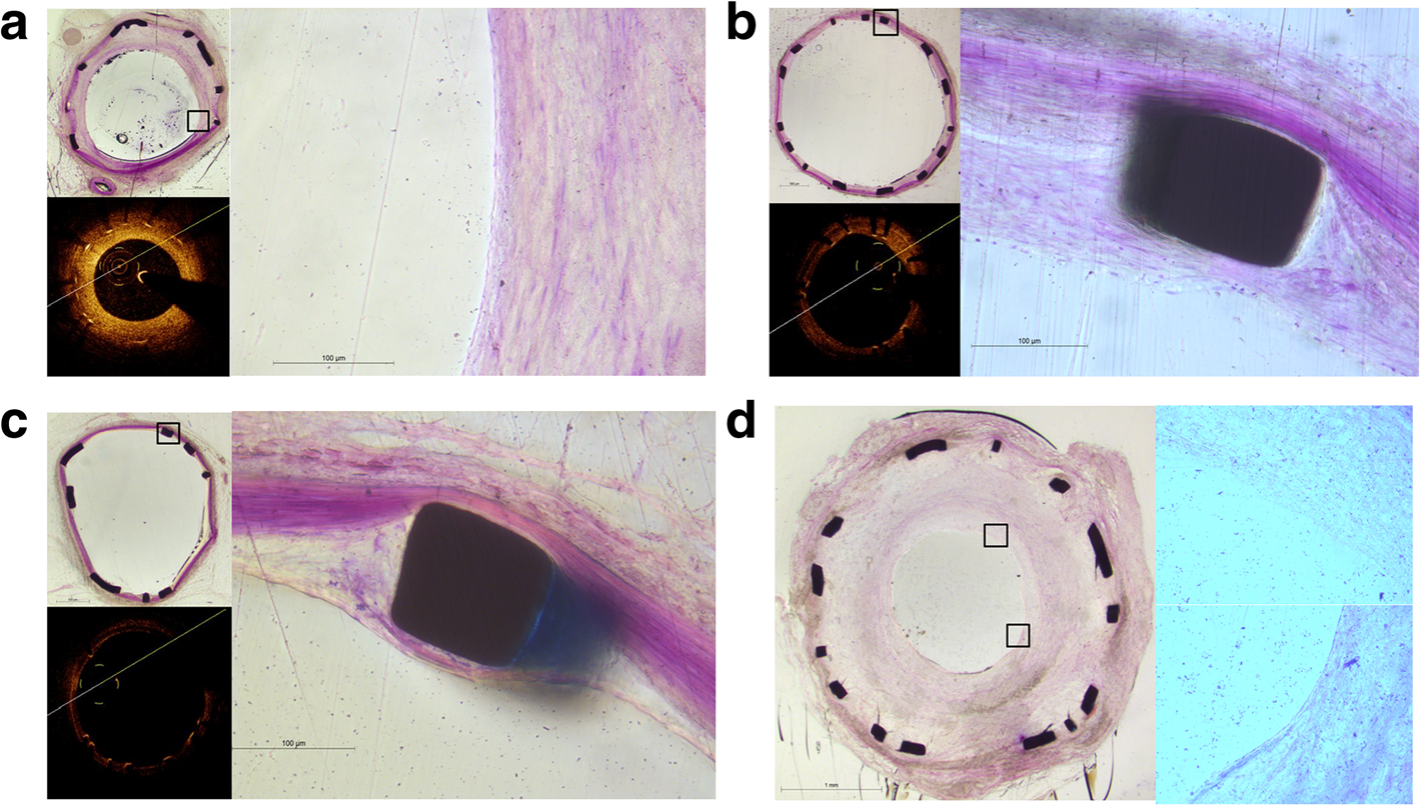
**a** The strut has been covered as observed by optical coherence tomography and at low magnification by hematoxylin and eosin (HE) staining. *Black rectangles* in the left image represent the area of the right image that the covering was endothelial cells in high magnification; **b** The strut has been covered as observed by optical coherence tomography and at low magnification by hematoxylin and eosin staining. *Black rectangles* in the *left* image represent the area of the *right* image that the covering was not endothelial cells in high magnification; **c** The strut has not been covered as observed by optical coherence tomography and at low magnification with hematoxylin and eosin staining. *Black rectangles* in the *left* image represent the area of the *right* image that the covering was not endothelial cells in high magnification; **d** The strut has been covered as observed at low magnification with hematoxylin and eosin staining, but *black rectangles* in the *left* image represent the area of the *right* image that the covering was not exactly the same

Our OCT results show that, at the 14-day time point, the strut coverage percentage in the PrES group was significantly higher than that observed in the SES group. Also, it had the highest percentage of EC coverage under SEM. However, such difference did not translate into mRNA and protein expression. At the 28-day time point, there was no significant difference between the groups with respect to strut coverage percentage, but it seemed to be higher in the BMS group than in the other groups, as observed by SEM. At high magnification, it compared with the PrES group, although the struts were almost completely covered in the BMS group with large part of the covering being only a layer of extracellular matrix, not EC (Fig. 4c). Conversely, although the struts were incompletely covered in the PrES group, the majority of the covering was by EC (Fig. 4d). There was also no obvious connection between the strut coverage observed by OCT and EC coverage assessed by HE staining at high magnification (Fig. 7).

There was no significant difference in the expression of both EC marker CD31 and EPC markers CD34 and CD133 in the mRNA and protein level between groups at all the four time points. In the histopathological analysis, there was also no significant difference in the endothelialization scores between groups at all the four time points. Overall, our results suggest that the local release of rapamycin at low concentration used in the SES and DDES groups did not significantly reduce the local expression of EC and EPC. These results are in agreement with those of previous in vitro studies [29, 30].

**Fig. 8:**
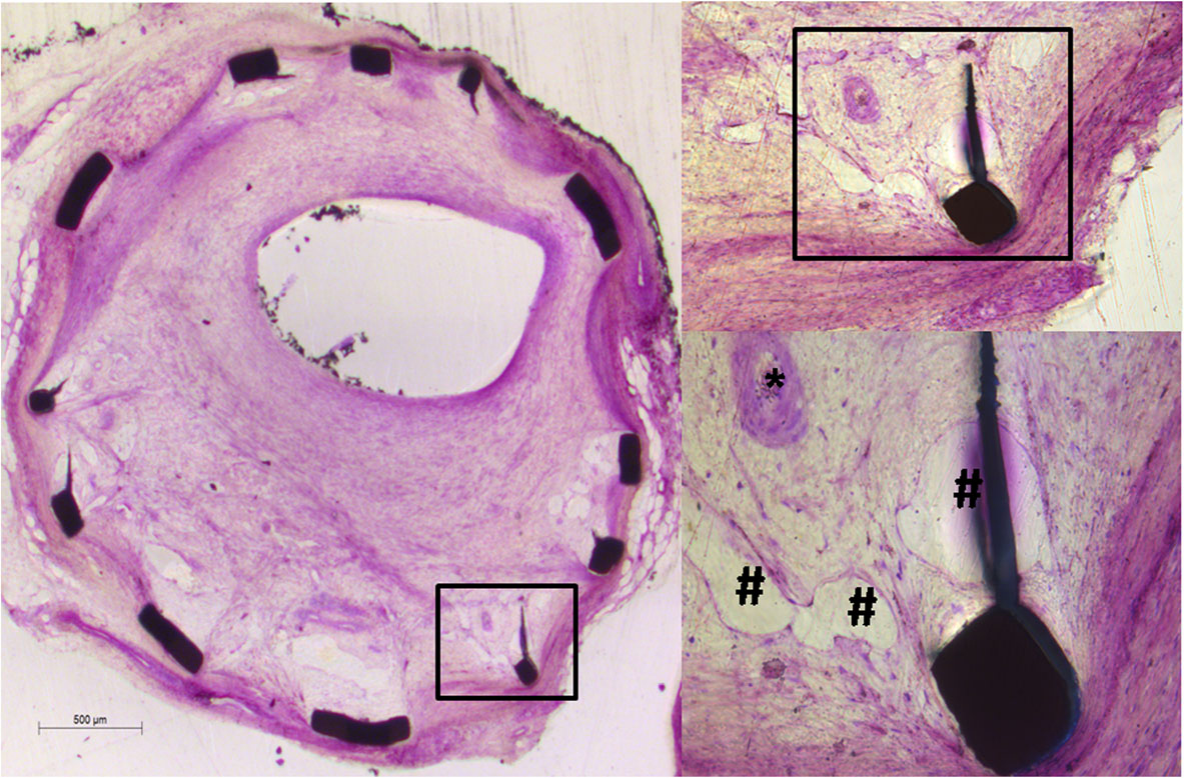
Angiogenesis in the neointima. # represents the venule in the neointima and * represents the arteriole in the neointima

Although rapamycin can inhibit the differentiation, proliferation, migration, adhesion and endothelial nitric oxide synthase expression of EPC [31], its effect on the homing of EPC at the site of the stented vessel segment might be different from previous studies. Also, the local release of probucol in the PrES and DDES groups showed a tendency to improve the short-term endothelialization when compared with the other groups in qualitative SEM analysis, although it was not detected in mRNA and protein level. Small sample sizes and the sampling method of the stented segments might have played a role. Lastly, at the time points of 90 and 180 days, obvious angiogenesis in the neointima was occasionally observed, resulting in individual higher injury scores as assessed by HE staining (Fig. 8). Although this phenomenon has never been reported in similar animal models, it is relatively common in studies of atherosclerotic plaques and in-stent neoatherosclerotic lesions [32, 33] and is often associated with late adverse events. In the present study, the development of this phenomenon interfered with the detection of the marker of endothelialization, likely due to the overlapping of endothelial cells and EPC at the lumen surface and angiogenesis. Consequently, this phenomenon could allow researchers to use this model for unstable atherosclerotic lesions and in-stent neoatherosclerotic lesions.

## Conclusion

Polymer-free dual drug-eluting stents are as safe as the commercially available BMS and SES, but they do not result in further improvement of the endothelialization of the stented porcine coronary arteries.

## Abbreviations

BMS: Bare-metal stent
CSR: Ratio of covered struts
DDES: Dual drug-eluting stent
EC: Endothelial cell
EPC: Endothelial progenitor cell
NA: Neointimal area
OCT: Optical coherence tomography
PCI: Percutaneous coronary intervention
PCR: Polymerase chain reaction
PrES: Probucol-eluting stent
QCA: Quantitative coronary angiography
SEM: Scanning electron microscope
SES: Sirolimus-eluting stents
SR: Stent restenosis
ST: Stent thrombosis
